# Effect of nanostructured silicon on surface enhanced Raman scattering†

**DOI:** 10.1039/c8ra00014j

**Published:** 2018-02-09

**Authors:** Gang Lu, Guilin Wang, Hai Li

**Affiliations:** Key Laboratory of Flexible Electronics (KLOFE), & Institute of Advanced Materials (IAM), Jiangsu National Synergetic Innovation Center for Advanced Materials (SICAM), Nanjing Tech University (NanjingTech) 30 South Puzhu Road Nanjing 211816 China iamhli@njteh.edu.cn

## Abstract

Non-metallic materials are often employed in SERS systems by forming composite structures with SERS-active metal materials. However, the role of the non-metallic structures in these composites and the effect of them on the SERS enhancement are still unclear. Herein, we studied the effect of silicon morphology on SERS enhancement on silver nanoparticles-coated different structured silicon surfaces. Our finding will help to further understand the SERS mechanism and pave the way for making more efficient SERS systems.

## Abstract 2

The surface morphology of non-metallic silicon has a big effect on the SERS enhancement of silver nanoparticle-coated silicon surfaces.

## Graphical abstract

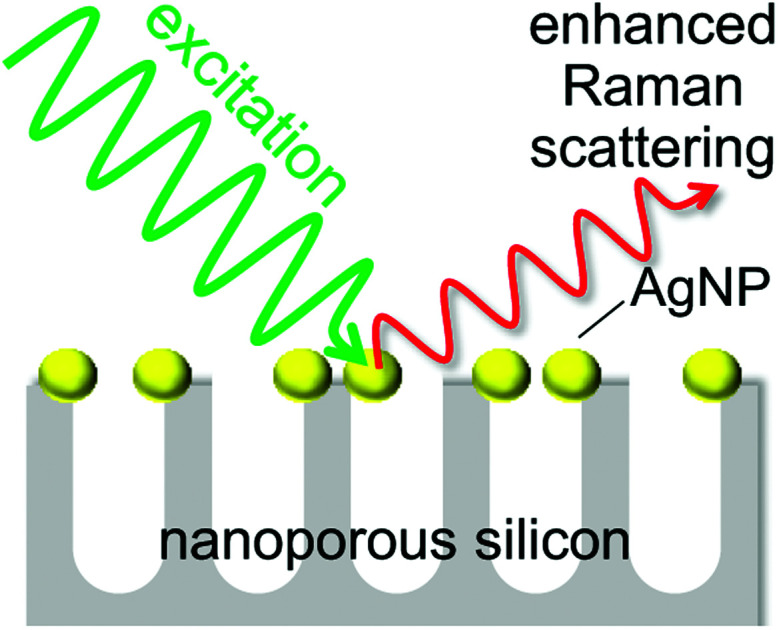

In past decades, increasing attention has been attracted to surface enhanced Raman scattering (SERS) due to the dramatically enhanced detection sensitivity of Raman scattering (down to single-molecule sensitivity). The Raman intensity of the molecules located in the vicinity of SERS nanostructures can be enhanced up to 10^10^ to 10^11^ times,^1,2^ largely extending the application of SERS in the fields of physics, chemistry and biology, *etc.*^3–9^ Metal materials are mainly employed in fabricating SERS structures, especially gold or silver ones for visible spectrum excitation.^10,11^ To date, many nanostructures have been reported that can enhance Raman scattering enormously, leading to so called Raman hot spots,^12–15^ including nanogaps,^16,17^ nanostars,^18^ nanotriangles and nanorods,^19^ mainly due to the introduction of a localized electromagnetic field under illumination. This kind of enhancement is referred to as the electromagnetic mechanism, which dominates the SERS enhancement in most cases.

Non-metallic structures can also contribute to Raman enhancement, although the enhancement factor is usually very low. It has been reported that Cu_2_O,^20^ TiO_2_ ^21^ and ZnS^22^ nanoparticles can enhance the Raman intensity of adsorbed molecules. Graphene has also been proved to be an efficient platform for Raman enhancement.^23^ Although non-metallic materials can be used directly for SERS applications, they are usually used by forming a composite with SERS-active metal material, where they act as supporting materials or borrow the SERS activity from the metallic Raman hot spots. It has been reported that SERS activity can be borrowed from SERS-active materials through ultrathin SERS-inactive transition metals (*e.g.*, Pt, Ni, Co and Pd)^24^ or dielectric (*e.g.*, SiO_2_, Al_2_O_3_)^25^ layer. Tian *et al.* reported the shell-isolated nanoparticle-enhanced Raman spectroscopy (SHINERS) by using the gold nanoparticles coated with ultra-thin silica or aluminum oxide shell.^26,27^ Raman enhancement can be achieved at the silica shell surface by borrowing the SERS activity from the gold core. However, the role of non-metallic structures in enhancing Raman scattering and the interactions between these two kinds of materials are still fuzzy.

Silicon nanostructures fabricated by catalytic etching method can be easily metalized with silver or gold by electroless deposition for SERS applications.^28–32^ However, up to now, only limited types of silicon nanostructures have been reported for SERS applications.^17,30,31^ In addition, the role of the nanostructured silicon surface in Raman enhancement is still unclear.

In this report, we fabricated SERS structures on two types of silicon surfaces, flat silicon and nanoporous silicon, by metallizing the silicon structure with silver nanoparticles (AgNPs). Compared to the fabricated SERS structure on flat silicon surface, the one fabricated on nanoporous silicon surface showed obvious enhancement on the Raman spectrum of adsorbed probe molecules. The effect of pore size and depth of nanoporous silicon on Raman enhancement was investigated in detail.

To investigate the role of silicon nanostructures in Raman enhancement, we compared the Raman spectra of probe molecules adsorbed on AgNPs-coated flat silicon and nanoporous silicon surfaces, respectively. The nanoporous silicon was fabricated by following a modified reported procedure (see details described in Experimental section and scheme shown in Fig. S1 in (ESI†)).^28^ Vertical nanopores were produced on silicon surface, and the pore size and pore depth can be easily tuned by varying the reaction parameters. Then a flat silicon and a nanoporous silicon substrates were both metallized with silver by immersing them into a mixed aqueous solution of AgNO_3_ and HF,^29,33^ forming AgNPs with size of 60 ± 30 nm. The as-prepared AgNPs-coated silicon surfaces (see scanning electron microscopy (SEM) images shown in Fig. S2 in ESI†) served as SERS-active substrates. After *p*-aminothiophenol (PATP) molecules (Raman probe) were adsorbed on the AgNPs-coated silicon structures, both AgNPs-coated substrates showed uniform and strong Raman enhancements (Fig. 1). On AgNPs-coated nanoporous silicon surface, the Raman bands of PATP molecules at 1076 and 1142 cm^−1^ are 4.2 and 7.4 times stronger compared to those on AgNPs-coated flat silicon surface (Fig. 1), respectively, demonstrating the vital role of silicon morphology in the obtained Raman enhancement. There are two widely accepted mechanism for SERS enhancement, electromagnetic mechanism and charge transfer mechanism.^1^ Both flat silicon and nanoporous silicon substrates are composed of same material and the only difference between them is the silicon morphology. Thus the charge transfer mechanism should contribute similar effect in both conditions. Moreover, both flat and nanoporous silicon surfaces were covered with a thin layer of silicon dioxide,^28,34^ which limit the charge transfer between AgNPs and silicon surface. This is also confirmed by the XPS measurement on the nanoporous silicon surface (Fig. S3 in ESI†). Therefore, the observed different enhancement may attribute to the electromagnetic mechanism, which will be discussed latter. In addition, the Raman enhancement is uniform over the whole substrate. This is probably due to the uniform coating of AgNPs on high-density silicon nanopore structures. The enhancement factor (EF) can be calculated by using the following equation, EF = (*I*_SERS_/*I*_bulk_)(*N*_bulk_/*N*_SERS_),^35^ where *I*_SERS_ and *I*_bulk_ represent the Raman intensities in SERS and bulk Raman measurements, respectively; *N*_SERS_ and *N*_bulk_ represent the number of probe molecules located in the excitation volume under these two conditions. For Raman band at 1076 cm^−1^ (represents a_1_ vibration mode of PATP,^11^ which sits at 1089 cm^−1^ for bulk,^36^ Fig. S4 in ESI†), the average EFs over the whole surface were calculated as 6.7 × 10^5^ and 2.8 × 10^6^ for SERS structures on flat silicon and nanoporous silicon, respectively. The strong Raman band at 1142 cm^−1^ indicates a chemical conversion from PATP to 4,4′-dimercaptoazobenzene (DMAB) upon light irradiation.^11^

**Fig. 1:**
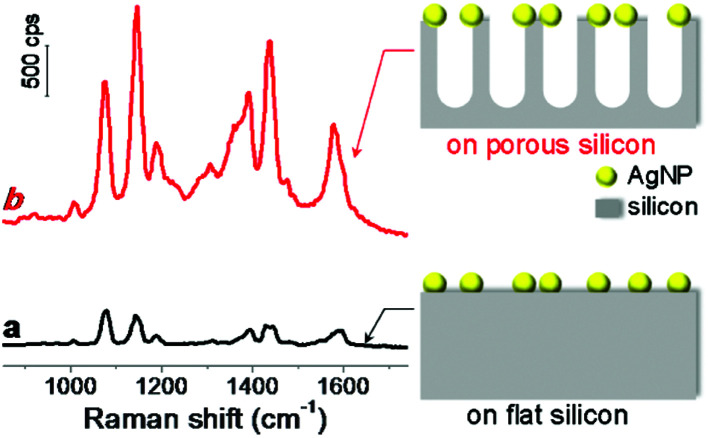
Raman spectra of PATP molecules adsorbed on the AgNPs-coated (a) flat silicon and (b) nanoporous silicon. A nanoporous silicon with pore depth of 220 nm was used here. The schemes at the bottom right and top right show the structures of the AgNPs-coated flat silicon surface and AgNPs-coated nanoporous silicon surface, respectively. The size of AgNPs was not drawn to scale.

As discussed, electromagnetic mechanism dominates the observed SERS enhancement. To confirm the role of silicon morphology, we did numerical simulation using the finite-difference time-domain (FDTD) method to investigate the localized electromagnetic field distributions on AgNPs-coated flat and nanoporous silicon surfaces. Note that, for the AgNPs-coated nanoporous silicon surface, many AgNPs sit on the edge of silicon nanopores (Fig. S2D in ESI†). In this case, the electromagnetic field around the AgNPs is more localized. Compared with the AgNPs-coated flat silicon, the electromagnetic field is five times more localized on the AgNPs-coated nanoporous silicon surface (Fig. 2), which in principal could introduce 25 times stronger Raman enhancement.^37^ However, in real case, only a proportional of the AgNPs locates on the edge of silicon nanopores and the shapes of the coated AgNPs are not exactly same with the ones we used in simulation, which explains the smaller SERS enhancement we observed on nanoporous silicon surface.

**Fig. 2:**
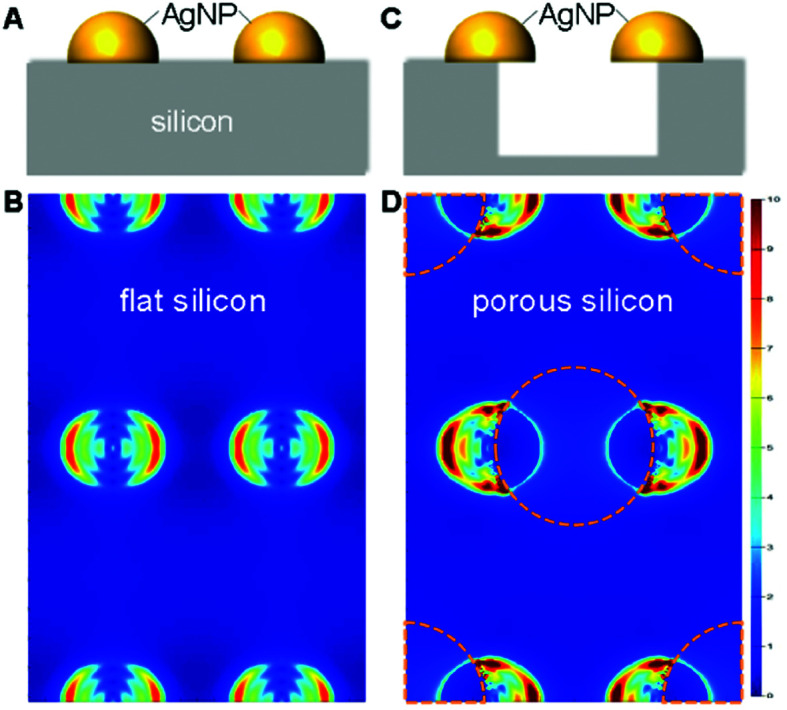
Schemes (side views) and FDTD simulations on the AgNPs-coated flat silicon (A and B) and nanoporous silicon (C and D). Dashed circles in (D) indicate the positions of silicon pores. The schemes in (A) and (C) were not drawn to scale.

As discussed above, the morphology of nanoporous silicon contributes to the enhanced Raman signal. By varying the pore size and pore depth of the nanoporous silicon, different Raman enhancement should be observed.

First, we studied the effect of pore depth of nanoporous silicon on Raman enhancement. The depth of silicon nanopores can be easily tuned by varying the period of catalytic etching of silicon. The Raman intensity of the probe molecules increased continuously with increased silicon nanopore depth (from 40 to 220 nm, Fig. 3). When PTAP molecules were adsorbed on the AgNPs-coated nanoporous silicon surface, the Raman intensity measured on silicon with 220 nm pore depth was increased about 2 times compared to that on silicon with 40 nm pore depth. Further increasing the pore depth to 900 nm, the Raman intensity dropped instead (Fig. 3). These results indicate the important role of the pore depth in Raman enhancement. FDTD simulations were carried out to investigate the mechanism behind (Fig. S5 in ESI†). As the pore depth increases, the electromagnetic field becomes more localized, which is consistent with the experimental data. However, the Raman intensity decreased on surface with very deep silicon nanopores (900 nm), which can be explained by the enhanced light trapping.^38,39^ In this case, part of the Raman scattering light cannot escape from the nanopores (confirmed by the dark black color of the sample, Fig. S6 in ESI†), leading to a weaker Raman signal. This can also be double confirmed by studying the Raman scattering from nanoporous silicon samples with AgNPs located at the bottom of the nanopores (discussed in ESI and Fig. S7†).

**Fig. 3:**
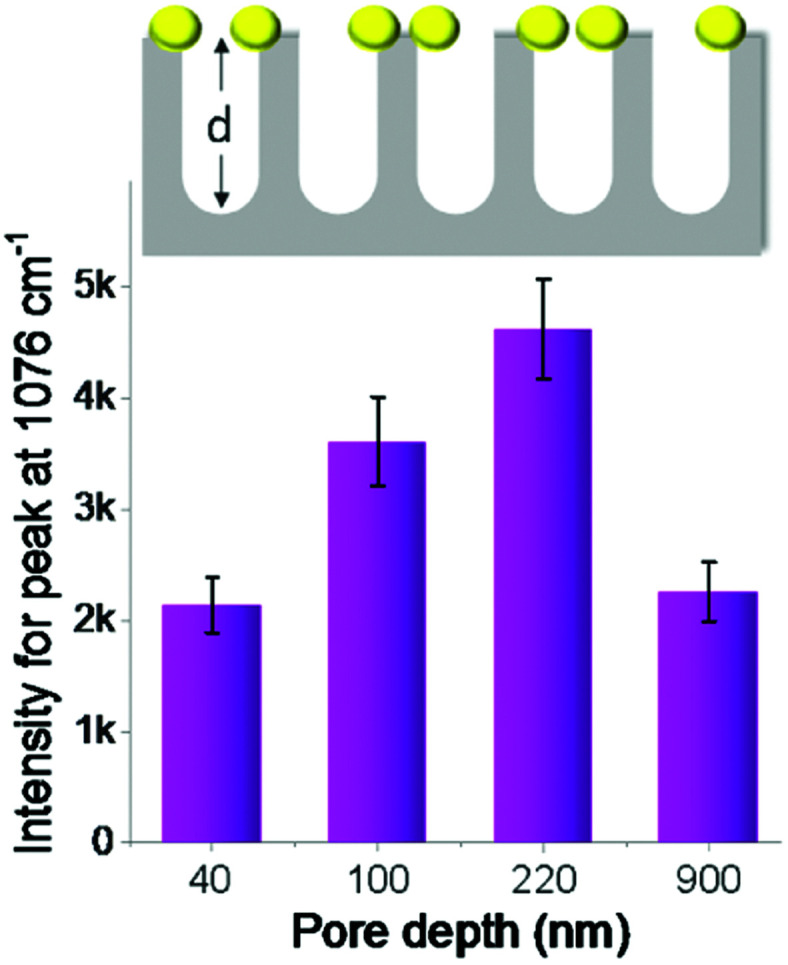
Raman intensity variation (peak at 1076 cm^−1^) on the AgNPs-coated nanoporous silicon (pore size of ∼40 nm was used) surface with four different depths. The scheme on top was not drawn to scale.

Second, the size of silicon nanopores also plays a role in the Raman enhancement. The pore size on silicon surface can be tuned by controlling the size of catalysts (AgNPs) deposited on silicon wafer (Fig. S1B in ESI†), whose size was replicated by the nanopores in subsequent catalytic etching process (Fig. S1C†). By varying the deposition time, nanoporous silicon samples with four different pores sizes, 31 ± 10, 41 ± 11, 80 ± 24 and 160 ± 50 (Fig. 4A–D), were fabricated, respectively. For the AgNPs-coated nanoporous silicon samples, the Raman intensity of probe molecules slightly changed while increasing the pore size (Fig. 4E), indicating a weak effect of pore size on Raman enhancement. As aforementioned discussion, the Raman enhancement is mainly contributed by the AgNPs that locate on the nanopore edges. Therefore, the Raman enhancement is strongly dependent on the perimeter of all the nanopores and the number of AgNPs that locate on the edge of silicon nanopores. While increasing the pore size, the perimeter of single pore increases. However, many pores are fused together, compensating the increase of the perimeter of single pore. Thus, the total perimeter of all nanopores does not change much when increasing the pore size. In this case, the amount of the AgNPs locating on the edge of silicon nanopores may not change too much, which may explain the less dependency of the pore size on SERS enhancement. To investigate the structure of the AgNPs-coated nanoporous silicon, we deposited gold nanoparticles (AuNPs) onto it. In this case, however, a stronger Raman scattering was observed due to the formation of AgNP–AuNP nanogaps and the enhancement varied on different sized silicon nanopores. When increasing the pore size from 31 ± 10 to 80 ± 24 nm, the Raman scattering became stronger. Further increasing the pore size to 160 ± 50 nm, the Raman intensity decreased. As known, two particles formed nanogap shows a more localized electromagnetic field when the polarization of incident light is parallel to the center to center axis of the two particles.^16,40^ Therefore, horizontal positioned two-particle nanogaps will give much stronger Raman enhancement. If the size of the silicon nanopores is too small, it is difficult for the AuNPs (13 nm in diameter) to enter the pore, limiting the number of AgNP–AuNP nanogaps that are horizontally positioned, and in turn limiting the Raman enhancement. While increasing the nanopore size, we have a better chance to form the ideally positioned AgNP–AuNP nanogaps to improve the Raman enhancement (Fig. S8 in ESI†). However, when the pore size is too big, only a small part of the nanostructures locates inside the excitation volume during Raman measurement, leading to a weaker Raman signal.

**Fig. 4:**
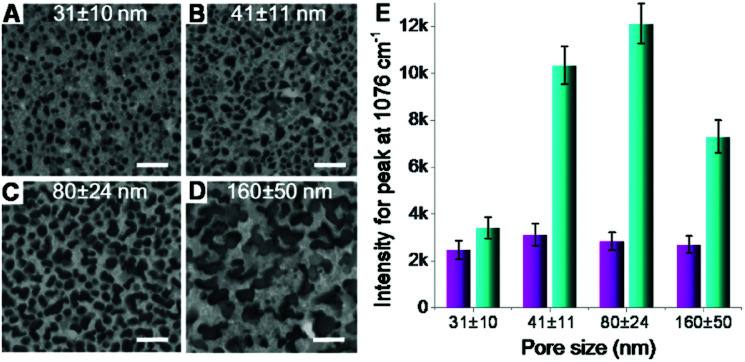
(A–D) Nanoporous silicon with different pore sizes obtained by varying the silver deposition time described in Fig. 1A and B. Scale bars = 200 nm. The pore depth here was set as 220 nm. (E) Raman intensity variation (peak at 1076 cm^−1^) on the AgNPs-coated nanoporous (four different pore sizes shown in A–D) silicon surface without (magenta bars) and with (cyan bars) the adsorption of AuNPs.

The Raman enhancement can also be affected by the size of the AgNPs coated on nanoporous silicon. The AgNP size can be tuned by varying the AgNP deposition time shown in Fig. S1E.† It has been reported that AgNPs with several tens of nanometers showed optimized plasmon resonance with excitation wavelength of 632.8 nm.^41^ In this work, the 40–75 nm AgNPs coated on nanoporous silicon show higher Raman enhancement than those with smaller or bigger AgNPs (Fig. S9†), since the size of these AgNPs fall into the optimized range for Raman enhancement, which is consistent with the reported work.

## Conclusions

In conclusion, by fabricating SERS structures on flat silicon and nanoporous silicon surfaces, we studied the effect of silicon morphology on observed Raman enhancements. It was found that the Raman signal of PTAP on nanoporous silicon surface is 4.2 to 7.4 times stronger than that on flat silicon surface. The Raman signal increased with increasing pore depth and reached maximum when pore depth was about 220 nm. While the pore size plays a weak role in the Raman enhancement. Our results demonstrate that the nanostructures of silicon affect the enhancement of nearby SERS structures. This finding will help us to understand the Raman enhancement from the metal–non-metal composite SERS systems and fabricate more efficient SERS substrates, which will promote the applications of SERS system in many fields.

## Experimental section

### Materials

Hydrogen fluoride (HF, 40%), hydrogen peroxide (H_2_O_2_, 30%), concentrated sulfuric acid (H_2_SO_4_, 98%), ethyl alcohol (95%), *p*-aminothiophenol (PATP) and silver nitrate (AgNO_3_) were purchased from Sigma-Aldrich Pte Ltd. Silicon (100) wafers were purchased from Bonda Technology Pte Ltd. All chemicals were used as received without further purification. Milli-Q water (Milli-Q System, Millipore, Billerica, MA, USA) was used in all experiments.

### Preparation of SERS substrates

After being sonicated in acetone for 10 min and rinsed with Milli-Q water, silicon (100) wafers were immersed into a freshly prepared piranha solution (*V*(H_2_SO_4_) : *V*(H_2_O_2_) = 7 : 3) at 100 °C for 30 min, followed by rinsing with Milli-Q water and drying with nitrogen gas. The cleaned silicon wafers were coated with silver nanoparticles (AgNPs) by immersing them in a mixed aqueous solution of AgNO_3_ (2 mM) and HF (1 wt%)^29,33^ for 15 s to 5 min (Fig. S1B in ESI†). The coated AgNPs served as catalyst in the subsequent catalytic etching of silicon, which was carried out in a mixed aqueous solution of H_2_O_2_ (0.6 wt%) and HF (5 wt%),^28,33^ forming high-density nanopores on silicon surface (Fig. S1C in ESI†). The as-fabricated nanoporous silicon were metalized with silver after being immersed in a mixed aqueous solution of AgNO_3_ (2 mM) and HF (1 wt%) for 15 s to 3 min (Fig. S1D in ESI†), forming a SERS-active AgNPs-coated nanoporous silicon substrate. The fabricated SERS substrate was immersed into a PATP ethanolic solution (1 mM) for 10 min to form self-assembled monolayers of PATP on the surface of AgNPs, followed by being thoroughly rinsed with ethanol and dried with nitrogen gas. For the adsorption of AuNPs, this substrate was immersed into a colloidal solution of 13 nm AuNPs for 10 min (Fig. S1E in ESI†). The obtained substrates were used for SERS measurements.

### Characterization of SERS substrates

Scanning electron microscopy (SEM) images were obtained by using a JEOL JSM-6340F field-emission scanning electron microscope at an accelerating voltage of 5 kV. The Raman measurement was carried out on a WITec alpha300 confocal Raman microscopy system with excitation line of 632.8 nm and an air-cooled charge coupled device (CCD) as the detector (WITec Instruments Corp, Germany). The laser power density at sample position was set as 10 kW cm^−2^, and integration time for each spectrum was 30 s. The Raman band of a silicon wafer at 520 cm^−1^ was used as a reference to calibrate the spectrometer.

## Conflicts of interest

There are no conflicts to declare.

## Supplementary Material

RA-008-C8RA00014J-s001
