# Supplementary material for: Effect of nanostructured silicon on surface enhanced Raman scattering
Source: RSC Adv. 2018 Feb 9;8(12):6629–33. doi: 10.1039/c8ra00014j (PMC9078225; doi:10.1039/c8ra00014j)
Supplement: RA-008-C8RA00014J-s001 [file RA-008-C8RA00014J-s001.pdf]

Electronic Supplementary Material (ESI) for RSC Advances.
This journal is © The Royal Society of Chemistry 2018

Supporting Information

Effect of Nanostructured Silicon on Surface Enhanced Raman Scattering

Gang Lu, Guilin Wang, Hai Li*

Key Laboratory of Flexible Electronics (KLOFE) and Institute of Advanced Materials (IAM), Jiangsu National Synergetic Innovation Center for Advanced Materials (SICAM), Nanjing Tech University (NanjingTech), 30 South Puzhu Road, Nanjing 211816, China

Corresponding Author

Hai Li: iamhli@njtech.edu.cn

Fabrication of nanoporous silicon

The SERS substrates were fabricated following the procedure as shown in Figure 1. First, nanoporous silicon was prepared by using a reported method.[1] Briefly, a silicon (100) wafer was deposited with AgNPs, electrolessly, by being immersed in an aqueous solution of $AgNO_3$ and HF (Figure S1A-B).[2, 3] Then these AgNPs serve as catalysts during the catalytic etching of silicon, forming nanoporous silicon, by immersing the wafer in an aqueous solution of H_2O_2 and HF (Figure S1C). After removing the silver catalysts with aqua regia solution (Figure S1D), the obtained nanoporous silicon wafer was coated again with AgNPs using the above-mentioned method, resulting in a SERS-active substrate (Figure S1E). The SERS performance of this AgNP-coated nanoporous silicon substrate was investigated after being coated with *p*-aminophenol (PATP, a Raman probe). The Raman enhancement can be further increased by adsorbing AuNPs, electrostatically, onto this SERS substrate (Figure S1F). Note that the whole fabrication process is simple and convenient, all conducted in aqueous solution and ambient condition. Moreover, the size and shape of the nanostructure can be easily tuned by varying the reaction time of silver coating and silicon etching, which will help us to utilize optimized SERS substrates easily.

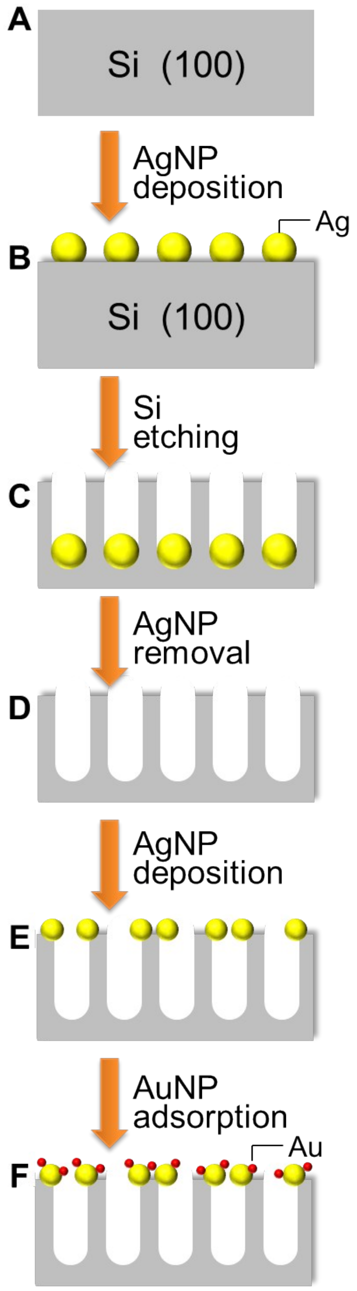

Figure S1. Schematic illustration of the fabrication procedure of nanoporous silicon-based SERS substrates. (A-B) AgNPs were deposited on silicon (100) wafer. (C) Nanoporous silicon formed *via* catalytic etching of Si wafer. (D) The AgNPs at the bottom of the pores were removed. (E) The formed nanoporous silicon in (D) were metalized with silver, which was used for SERS measurement. (F) AuNPs were adsorbed on sample (E) *via* the electrostatic interaction.

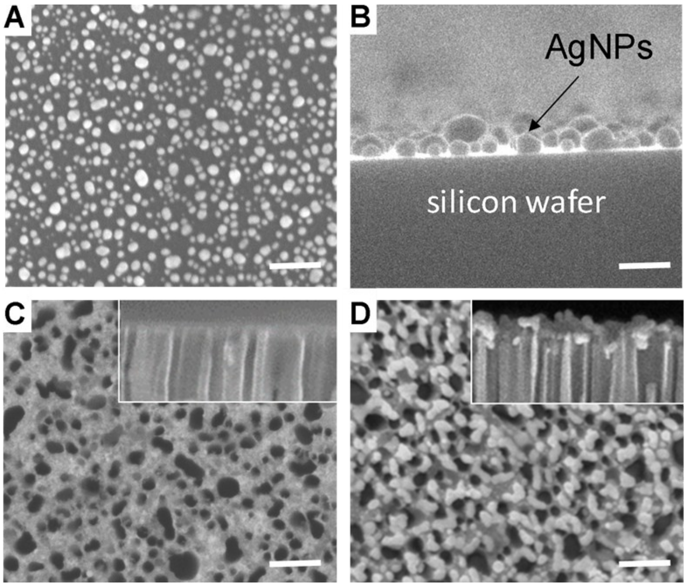

Figure S2. (A-B) Top and section view images of typical AgNP-coated flat silicon surface. (C-D) typical SEM images of the catalytic etching-induced nanoporous silicon and AgNP-coated nanoporous silicon. surface. Scale bar = 200 nm. The images of cross section view were shown in insets in (C-D).

The surface oxidation state of the silicon surfaces

In a previous report,[1] it has been clarified that the fabrication of nanoporous silicon using catalytic etching method involves the metal-induced (either by Ag or Au) excessive local oxidation and dissolution of silicon. So there should be oxidation layer on silicon surface. We also did XPS measurement on the fabricated nanoporous silicon surface. In Figure S3, clear O 1s, Si 2s and Si 2p peaks can be observed, indicating the oxidation of silicon on surface. The atomic concentration of silicon is 36.4% and atomic concentration of oxygen is 34.2%, giving a Si:O atomic ratio of 1:0.94 (the ratio is 1:2 for SiO_2), telling us that silicon is partially oxidized on surface. We also tried to etch the surface oxide layer with hydro fluoric acid, and the structure remains unchanged, indicating the very thin layer of oxide. Therefore, the obtained

nanoporous silicon in this report is made of silicon with surface partially oxidized. We also would like to point out that silicon surface is easily oxidized in ambient condition, which has been demonstrated in literature.[4] So there is an oxide layer even on un-etched silicon surface.

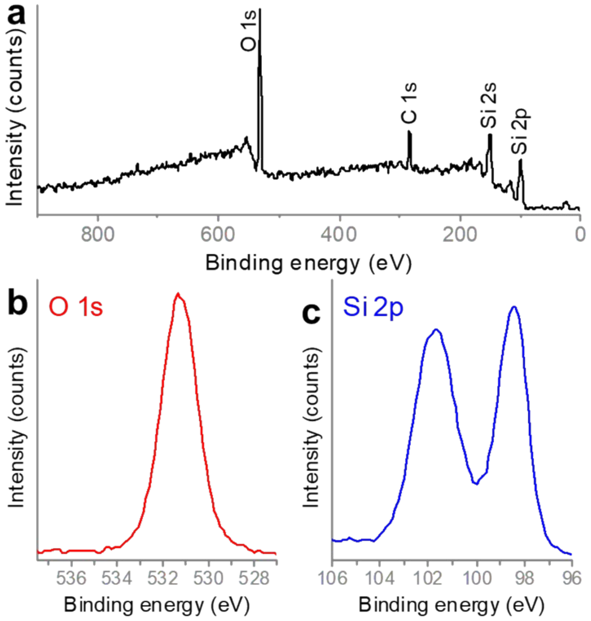

Figure S3. (a) XPS spectrum of the nanoporous silicon surface. (b-c) Magnified XPS spectra of O 1s and Si 2p shown in (a), respectively.

Table S1. Atomic concentration of elements.

**Atom**	**C**	**Si**	**O**
**Concentration**	29.4	36.4	34.2

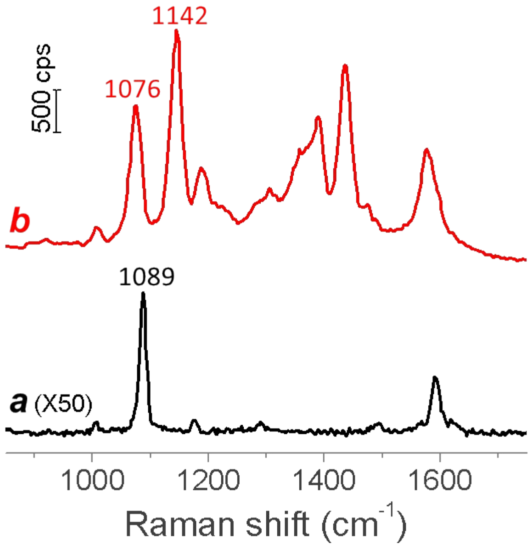

Figure S4. Raman spectra of bulk PATP on flat silicon substrate (curve *a* was 50-fold magnified) and the PATP molecules adsorbed on AgNP-coated nanoporous silicon (curve *b*).

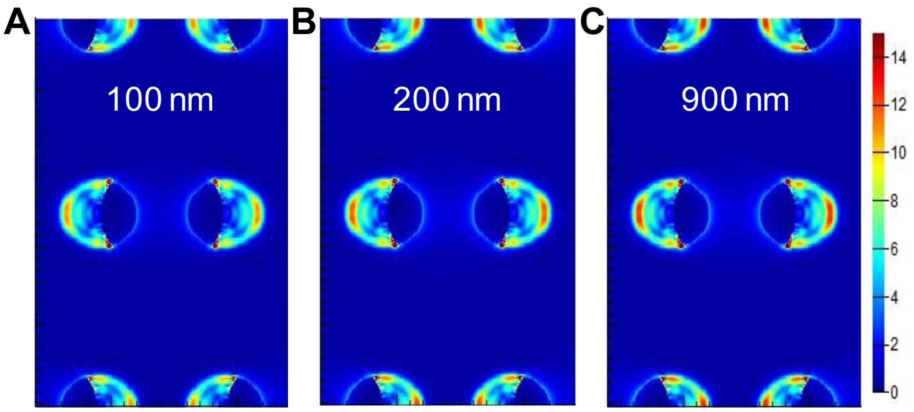

Figure S5. FDTD simulations on AgNP-coated nanoporous silicon with different depths of 100 nm (A), 200 nm (B) and 900 nm (C).

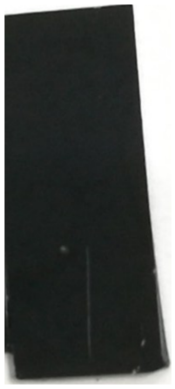

Figure S6. Photograph of the 900 nm deep nanoporous silicon.

Enhancement factor (EF) calculation

We calculate the EF by using an equation, $EF=(I_{SERS}/I_{bulk})\cdot(N_{bulk}/N_{SERS})$, demonstrated in our previous reports.[5, 6] To answer the question from reviewer, N_{bulk} represents the number of probe molecules in excitation volume during bulk measurement. Usually,

bulk PATP was deposited on solid substrate, and then excited with a focused laser beam. The excitation volume can be calculated from the Abbe's equation, $d_{xy} = \frac{1.22\lambda}{NA}$, $d_z = \frac{4\lambda \cdot \eta}{NA^2}$. Where d_{xy} represents the spot size in *xy* direction, d_z represents the spot size in *z* direction. NA is the numerical aperture of the objective, which is 0.6 for the objective we used. Note that the penetration depth of the laser in bulk PATP is bigger than d_z. So the excitation volume here is equal to the volume in which the probe molecules were excited in bulk measurement. This volume can be calculated by the equation $V = \pi {d_{xy}}^2 d_z$ and the density of bulk PATP is known as ~1.18 g cm³. Then it is easy to calculate the number of molecules in this excitation volume. N_{SERS} represents the number of molecules inside excitation volume in SERS measurement condition. We assume the nanoporous silicon was covered with a monolayer of PATP molecules, resulting a monolayer with height of roughly 0.5 nm. The excitation spot size can be obtained from the Abbe's equation, $d_x = \frac{1.22\lambda}{NA}$. Then the volume of the excited molecules can be calculated. Therefore, the number of the molecules in this volume can be calculated accordingly.

The influence of pore depth on Raman enhancement

To double confirm the influence of pore depth, we prepared Raman hot spots at the bottom of silicon nanopores. As mentioned, during the catalytic etching of silicon, AgNPs act as catalysts and move to the bottom of the nanopores along with the etching of silicon, which can also be used as Raman hot spots. After functionalized with Raman probe molecules (PATP), this structure was used to investigate the depth

effect of silicon nanopores (Figure S7). While increasing the depth of silicon nanopores, the Raman intensity kept stable firstly (depth < 450 nm), and then decreased dramatically when the depth goes up to 450 nm. When further increases the depth to 900 nm, Raman signal can hardly be observed. This tells us that Raman scattered light can escape from the nanopore when the depth is less than 450 nm, however, the Raman scattered light is trapped inside the silicon nanopores when the nanopore is too deep. This is also confirmed by the dark black color of the 900 nm deep nanoporous silicon surface, implying the efficient trapping of light.

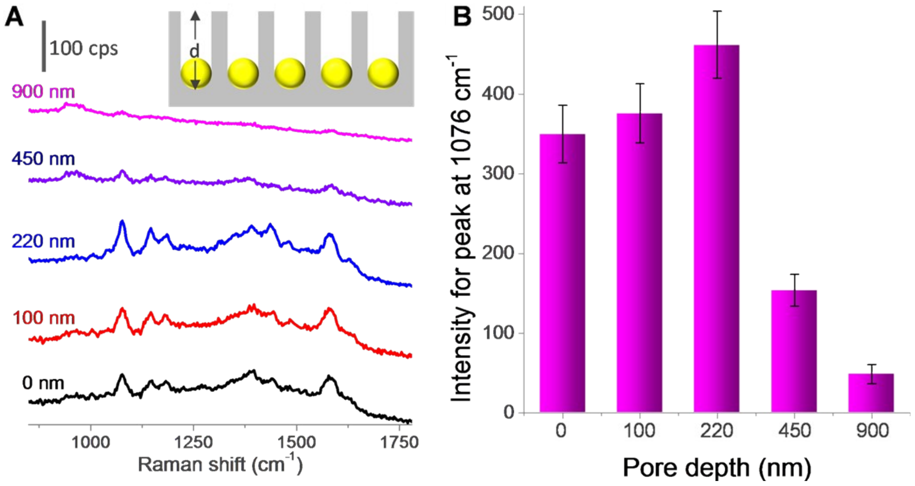

Figure S7. Raman spectra of PATP molecules from the AgNPs that locates at the bottom of silicon nanopores (pore size of ~40 nm was used) with different depths.

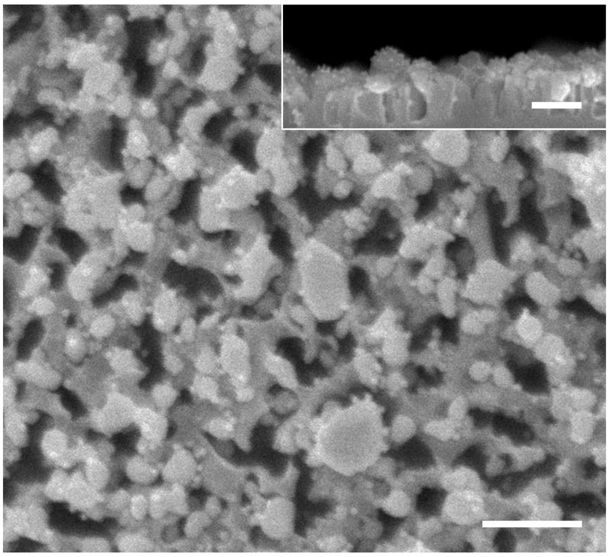

Figure S8. Typical SEM image of the AgNP-coated nanoporous silicon adsorbed with AuNPs. Inset image at top right shows the side view of the structure. Scale bars = 200 nm.

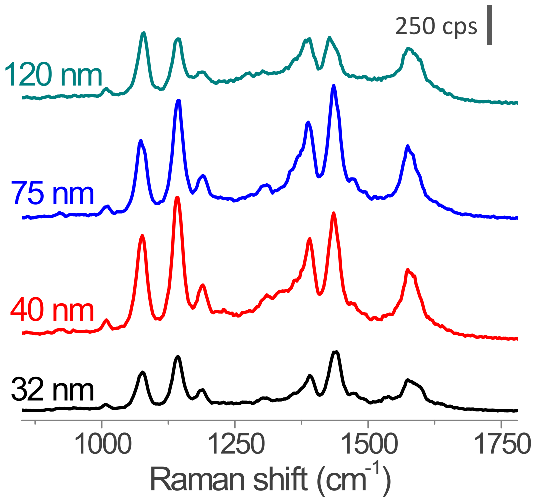

Figure S9. Raman spectra of PATP molecules on different sized (Ag deposition time in step Figure S1E was shown) AgNP-coated nanoporous.

Supporting References:

1. Peng, K. Q.; Hu, J. J.; Yan, Y. J.; Wu, Y.; Fang, H.; Xu, Y.; Lee, S. T.; Zhu, J., Fabrication of single-crystalline silicon nanowires by scratching a silicon surface with catalytic metal particles. *Adv. Funct. Mater.* 2006, 16, 387-394.
2. Peng, K. Q.; Lu, A. J.; Zhang, R. Q.; Lee, S. T., Motility of Metal Nanoparticles in Silicon and Induced Anisotropic Silicon Etching. *Adv. Funct. Mater.* 2008, 18, 3026-3035.
3. Qiu, T.; Chu, P. K., Self-selective electroless plating: An approach for fabrication of functional 1D nanomaterials. *Mater. Sci. Eng. R Rep.* 2008, 61, 59-77.
4. Ryckman, J. D.; Reed, R. A.; Weller, R. A.; Fleetwood, D. M.; Weiss, S. M., Enhanced room temperature oxidation in silicon and porous silicon under 10 keV x-ray irradiation. *J. Appl. Phys.* 2010, 108, 113528.
5. Lu, G.; Li, H.; Zhang, H., Nanoparticle-Coated PDMS Elastomers for Enhancement of Raman Scattering. *Chem. Commun.* 2011, 47, 8560-8562.
6. Lu, G.; Li, H.; Wu, S. X.; Chen, P.; Zhang, H., High-density metallic nanogaps fabricated on solid substrates used for surface enhanced Raman scattering. *Nanoscale* 2012, 4, 860-863.
